# New Azaphilones from *Nigrospora oryzae* Co-Cultured with *Beauveria bassiana*

**DOI:** 10.3390/molecules23071816

**Published:** 2018-07-21

**Authors:** Zhuo-Xi Zhang, Xue-Qiong Yang, Qing-Yan Zhou, Bang-Yan Wang, Ming Hu, Ya-Bin Yang, Hao Zhou, Zhong-Tao Ding

**Affiliations:** Functional Molecules Analysis and Biotransformation Key Laboratory of Universities in Yunnan Province, School of Chemical Science and Technology, Yunnan University, 2st Cuihu North Road, Kunming 650091, China; 13278750367@163.com (Z.-X.Z.); yangxq@ynu.edu.cn (X.-Q.Y.); 13187884610@163.com (Q.-Y.Z.); gjk201113409218@163.com (B.-Y.W.); Hm1720553468@ynu.edu.cn (M.H.); zhouhaoa@163.com (H.Z.)

**Keywords:** *Nigrospora oryzae*, *Beauveria bassiana*, co-culture, azaphilone, antifungal selectivity, NO inhibitory activity

## Abstract

In this study, the co-culture of *Nigrospora oryzae* and *Beauveria bassiana*, the endophytes in the seeds of *Dendrobium officinale*, were examined for metabolite diversity. Five new azaphilones were isolated, and their structures were determined by spectral analysis. In terms of azaphilones, compound **2** had an unprecedented skeleton, with a bicyclic oxygen bridge. The antifungal selectivities of the metabolite produced by *N. oryzae* against its co-culture fungus, *B. bassiana*, and common pathogens exhibited competitive interaction in this mix-culture. Compounds **1** and **2** showed obvious nitric oxide (NO) inhibitory activity with ratios of 37%, and 39%, respectively, at a concentration of 50 μM.

## 1. Introduction

**Figure 1:**
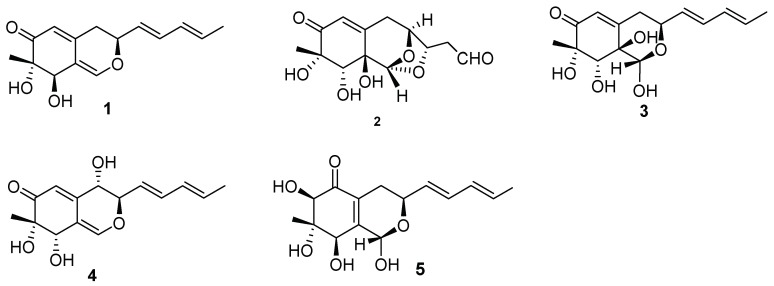
Structures of compounds **1**–**5**.

In the last ten years, several methods have been developed to aid in the activation of the cryptic biosynthetic pathways of microbial metabolites. One of these approaches is microorganism co-culture, involving the cultivation of two or more microorganisms in the same confined environment [1,2]. Microorganism co-culture can be achieved in either solid or liquid media, and has recently been both increasingly and extensively used to study natural interactions and discover new bioactive metabolites [3,4,5]. In this study, we investigated the metabolic mechanism of compounds produced by *Nigrospora oryzae* co-cultured with *Beauveria bassiana*. Five new azaphilones, **1**–**5**, were obtained from co-culture of *N. oryzae* and *B. bassiana*, as shown in Figure 1. The antifungal activities of the metabolites produced by *N. oryzae* against its corresponding co-culture fungus, *B. bassiana*, were screened. The competitive interactions of co-cultured fungi induced the selectivity of the antifungal activities through the production of diverse metabolite structures. The compounds were also screened for their inhibition of acetylcholinesterase (AChE), nitric oxide (NO), pancreas lipase, tyrosinase and cytotoxicity.

## 2. Results and Discussion

**Figure 2:**
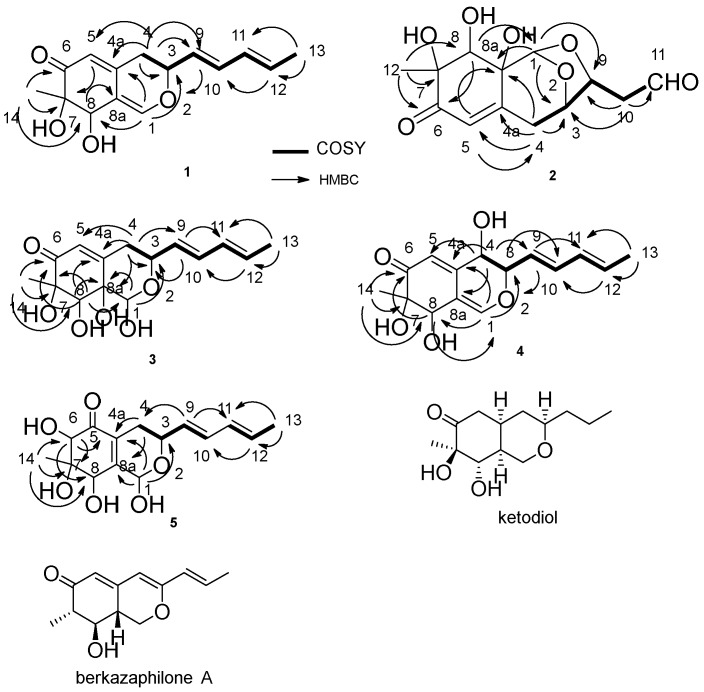
Correlation spectroscopy (COSY) and heteronuclear multiple bond correlation (HMBC) correlations of compounds **1**–**5**, and structures of ketodiol and berkazaphilone A.

The molecular formula of nigbeauvin A (**1**) was determined as C_15_H_18_O_4_ from high resolution electrospray ionization mass spectrum (HRESIMS) analysis. ^1^H and ^13^C NMR spectroscopic analyses, including Distortionless Enhancement by Polarization Transfer (DEPT) clearly showed two methyls, one methylene, eight methines, two olefinic quaternary carbons, one quaternary carbon and one carbonyl carbon, the sum of which indicated the skeleton of azaphilone [6]. correlation spectroscopy (COSY) correlations of H-4/H-3/H-9/H-10/H-11/H-12/H-13, and heteronuclear multiple bond correlation (HMBC) correlations from: H-14 to C-6, C-7 and C-8; H-4 to C-3, C-4a, C-5 and C-9; H-1 to C-3 and C-4a; H-12 to C-10; and H-13 to C-11 and C-12, confirmed this structure (Figure 2). The OH connected to C-8 was determined by the HMBC correlations from H-1 to C-8 and H-8 to C-8a (Figure 2). The relative configuration of the stereocenters between C-7 and C-8 in compound **1** was determined to be the same as that of falconensin by comparing the NMR with those of ketodiol (Figure 2) [7]. The nuclear overhauser enhancement spectroscopy (NOESY) correlation between H-3/H-8 confirmed the relative configuration between them. The absolute configuration of C-7 was determined as S by comparing the circular dichroism (CD) spectrum with those of daldinins [8]. The CD spectrum of **1** showed negative first (350 nm) and positive second (240 nm) Cotton effects. The calculated electronics circular dichroism (ECD) also exhibited a negative effect (346 nm).

The molecular formula of nigbeauvin B (**2**) was determined as C_13_H_16_O_7_ from HRESIMS analysis. ^1^H and ^13^C NMR spectroscopic analyses clearly showed one methyl, two methylenes, five methines, one olefinic quaternary carbon, two quaternary carbons, one aldehyde group, and one carbonyl carbon, which indicated a skeleton similar to that of compound **1**. The COSY correlations of H-4/H-3/H-9/H-10/H-11, and HMBC correlations from: H-10 to C-3, C-9 and C-11; H-1 to C-3, C-9 and C-8a; H-4 to C-3, C-4a, C-5 and C-8a; H-5 to C-8a; and H-12 to C-6, C-7 and C-8, confirmed this structure. The OH connected to C-8 was determined by the HMBC correlations from H-8 to C-1 (Figure 2). With respect to azaphilones, this compound had an unprecedented skeleton, with a bicyclic oxygen bridge. The relative configuration of this compound was determined by the NOESY correlations of H-1/H-8, H-8/H-12 and H-3/H-9, and the NMR, by comparison with those of berkazaphilone A (Figure 2) [9]. The absolute configuration of C-7 was determined as S by the CD spectrum, which demonstrated negative (348 nm) and positive (237 nm) Cotton effects.

Compounds **3** and **4** were isolated as a mixture in a 1:1 ratio. The molecular formula of nigbeauvin C, D (**3**, **4**) was determined from HRESIMS analysis as C_15_H_20_O_6_ for **3** and C_15_H_18_O_5_ for **4**. The ^1^H and ^13^C NMR spectroscopic analyses clearly showed a skeleton similar to that of compound **1**. The spectra of both compounds were almost identical, except for the NMR signals, due to part of the hydrogenated pyran. The hydrogenated pyran of **3** was confirmed by the COSY correlations of H-4/H-3/H-9/H-10/H-11/H-12/H-13, and HMBC correlations from H-4 to C-3, C-4a, C-5, C-8a and C-9, and H-1 to C-3 and C-8a. The remaining part of the structure was determined by the HMBC correlations from: H-9 to C-3 and C-11; H-13 to C-11 and C-12; H-14 to C-6, C-7 and C-8; and H-8 to C-1, C-6, C-7 and C-8a. The OH connected to C-8 was determined by the HMBC correlations from H-8 to C-1 (Figure 2). The hydrogenated pyran of **4** was confirmed by the COSY correlations of H-4/H-3/H-9/H-10/H-11/H-12/H-13, and HMBC correlations from H-4 to C-4a, C-5 and C-9, and H-1 to C-3 and C-8. The remainder of the structure was also determined by the HMBC correlations from: H-9 to C-3 and C-11; H-13 to C-11 and C-12; H-14 to C-6, C-7 and C-8; and H-8 to C-6. The OH connected to C-8 was determined by the HMBC correlations from H-1 to C-8 and H-8 to C-1 (Figure 2). The relative configuration of **3** was confirmed by the NOESY correlations between H-1/H-8/H-14 and H-1/H-9. The relative configuration of **4** was determined by the NOESY correlations between H-4/H-9 and H-4/H-8/H-14. By comparing the NMR with those of **1** and **2**, the absolute configurations of C-7 in compounds **3** and **4** were determined to be the same as those of compounds **1**–**2**, i.e., the S configuration.

The molecular formula of nigbeauvin E (**5**) was determined as C_15_H_20_O_6_ from HRESIMS analysis. ^1^H and ^13^C NMR spectroscopic analyses clearly showed a skeleton similar to the other new compounds. The COSY correlations of H-4/H-3/H-9/H-10/H-11/H-12/H-13, and HMBC correlations from: H-1 to C-3, C-4a and C-8a; H-4 to C-4a and C-8a; H-14 to C-6, C-7 and C-8; and H-6 to C-5, C-7 and C-8, confirmed this structure (Figure 2). The relative configuration of **5** at C-6 and C-8 was confirmed by the NOESY correlations between H-6/H-8. The absolute configuration of **5** at C-7 was determined as S by CD spectrum, with a negative Cotton effect (342 nm).

To investigate the chemical interactions of the co-culture, *N. oryzae*-*B. bassiana*, the biogenesis of these metabolites was studied. There has been little research conducted on azaphilone isolation from *Nigrospora*, but a similar skeleton, such as pulvilloric acid-type azaphilone, has been regularly found as anthraquinone in *N. oryzae* [10]. Therefore, pulvilloric acid-type azaphilones are produced by *N. oryzae*. *B. bassiana* had no metabolite by itself in the mixed culture (*N. oryzae*-*B. bassiana*) because of lower growth compared with that of *N. oryzae* in the plate culture. The antifungal selectivities of the metabolites produced by *N. oryzae* against the co-cultured fungus and other pathogens, such as *Monilia albican* and *Bacillus subtilis*, showed competitive interactions. It was found that nigbeauvin A showed selectivity of antifungal activities against the co-cultured fungus, *B. bassiana*, with minimum inhibitory concentrations (MICs) of 128 μg/mL and 512 μg/mL observed against *N. oryzae*. Thus, the metabolic mechanism of mix-culture—for the benefit of survival of different fungi—can generate chemical diversity. Nigbeauvin A also exhibited the antibacterial activity against *B. subtilis* with MIC of 128 μg/mL, and no antifungal activity against *M. albican* with MIC >512 μg/mL. The inhibitory activities of compounds **1** and **2** against NO, AChE, tumor cells, porcine pancreas lipase (PPL) and tyrosinase were also investigated. Compounds **1** and **2** showed inhibitory activity against NO production with ratios at 37% and 39%, respectively, at a concentration of 50 μM. NG-monomethyl-L-arginine (L-NMMA) was used as the positive control with an inhibition ratio of 50.83 ± 0.66% at a concentration of 50 μM. Compounds **1** and **2** exhibited no obvious activity against AChE (inhibition ratio <10% at 50 μM), tumor cells (inhibition ratio <10% at 40 μM), PPL (inhibition ratio <10% at 50 μM), or tyrosinase (inhibition ratio <10% at 100 μM).

## 3. Materials and Methods

### 3.1. General Experimental Procedure

Silica gel (200–300 mesh; Qingdao Marine Chemical Group Co., Qingdao, China), Lichroprep RP-18 (Beijing Greenherbs and Technology and Development Co., Beijing, China) and Sephadex LH-20 (GE Healthcare Co., Buckinghamshire, UK), were used for column chromatography. 1D and 2D NMR spectra were obtained on Bruker AVANCE 400, 500 and 600 MHz NMR instruments (Bruker Co., Karlsruhe, Germany). MS spectra were recorded with Agilent G3250AA (Agilent, Santa Clara, CA, USA) and AutoSpec Premier P776 spectrometers (Waters, Milford, MA, USA). Optical rotations were obtained on a Jasco P-1020 polarimeter. Circular dichroism spectra were obtained in an Applied Photophysics Chirascan spectrometer.

### 3.2. Biological Material and Mixed Cultivation of Fungal Strains

The fungi were isolated from seeds of *Dendrobium officinale* from Wenshan, Yunnan Province. The species were identified as *N. oryzae*, and *B. bassiana* on the basis of morphological and genetic Internal Transcribed Spacer (ITS) characteristics. Voucher specimens were deposited at the School of Chemical Science and Technology, Yunnan University. *N. oryzae* and *B. bassiana* were maintained on Potato Dextrose Agar (PDA) medium (peeled and cut potato 200 g/L, glucose 20 g/L, agar 15 g/L). The mixed fermentation of *N. oryzae*-*B. bassiana* was cultured in 0.5 L Erlenmeyer flasks containing 120 mL of potato dextrose broth (PDB, potato infusion of 200 g fresh potato, dextrose 15 g, distilled water 1.0 L, pH 7.0), at 150 rpm and 28 °C for 3 days for seed culture. Each 20–25 mL of seed culture was transferred into a 1.0 L Erlenmeyer flask containing 200 mL of PDB, and incubated at 150 rpm and 28 °C for 7 days.

### 3.3. Extraction and Isolation of Compounds

The resulting mixed cultures of *N. oryzae*-*B. bassiana* (30 L) were centrifuged to separate the mycelia from the supernatant. The supernatant was exhaustively extracted with EtOAc, yielding an extract (10 g, *N. oryzae*-*B. bassiana*). The mycelia were then extracted three times with acetone for 3 days each time. The acetone was removed under vacuum, and the resulting aqueous layer was extracted three times with an equal volume of EtOAc, to yield a crude extract (15 g). The extracts of the fermentation broth and the mycelia were combined after thin layer chromatography (TLC) analyses. The residue (25 g) was first subjected to column chromatography (silica gel, CHCl_3_/MeOH 100:0, 50:1, 30:1, 10:1 and 3:1 (*v*/*v*)) to afford fractions 1–7. Fr. 2 was fractioned by a reversed-phase chromatography gradient, eluted with MeOH-H_2_O (50%), to afford compound **1** (72 mg). Fr. 2 was isolated on a Lichroprep RP-18 column with MeOH/H_2_O (30%, *v*/*v*) and further purified on a silica gel column with a CHCl_3_/MeOH gradient (30:1), to afford compound **2** (2.3 mg). Fr. 3 was fractioned by a Lichroprep RP-18 column with MeOH-H_2_O (10–50%) and a silica gel column with a CHCl_3_/MeOH gradient (50:1), to afford compounds **3** and **4** (19 mg), and compound **5** (2.2 mg).

Compound **1**: ${\left[\alpha \right]}_{D}^{22}$-39.7 (c 0.4, MeOH). HRESIMS *m*/*z*: 285.1122 [M + Na]^+^, calcd for C_15_H_18_O_4_Na: 285.1103. ^1^H-NMR (CDCl_3_, 500 MHz) and ^13^C-NMR (CDCl_3_, 125 MHz) in Table 1 and Supplementary Materials.

Compound **2**: ${\left[\alpha \right]}_{D}^{22}$-42.6 (c 0.2, MeOH). HRESIMS *m*/*z*: 307.0791 [M + Na]^+^, calcd for C_13_H_16_O_7_Na: 307.0794. ^1^H-NMR (acetone, 600 MHz) and ^13^C-NMR (acetone, 150 MHz) in Table 1 and Supplementary Materials.

Compounds **3**, **4**: HRESIMS *m*/*z*: 319.1161 [M + Na]^+^ for **3**, calcd for C_15_H_20_O_6_Na: 319.1158; HRESIMS *m*/*z*: 301.1054 for **4**, calcd for C_15_H_18_O_5_Na: 301.1052. ^1^H-NMR (CD_3_OD, 500 MHz) and ^13^C-NMR (CD_3_OD, 125 MHz) in Table 1 and Table 2 and Supplementary Materials.

Compound **5**: ${\left[\alpha \right]}_{D}^{22}$-13.2 (c 0.2, MeOH). HRESIMS *m*/*z*: 319.1153 [M + Na]^+^ for **5**, calcd for C_15_H_20_O_6_Na: 319.1158. ^1^H-NMR (CD_3_OD, 600 MHz) and ^13^C-NMR (CD_3_OD, 150 MHz) in Table 2 and Supplementary Materials.

### 3.4. Bioactive Assay

In the in vitro antimicrobial test, PDB was used as an incubation medium for fungi and Luria-Bertani (LB) medium was used for bacteria. The final volume of each well was 100 μL. Aliquots (5 μL) of the metabolite solutions in dimethyl sulfoxide (DMSO) were added into 96-well sterilized microplates, and their final concentrations ranged from 1 to 512 μg/mL using a twofold serial dilution method. Spore suspensions (5 μL) of *N. oryzae*, *B. bassiana*, *B. subtilis*, and *M. albicans* were inoculated in each well. The wells containing pathogenic fungi and bacteria suspensions, DMSO and the incubation medium were employed as negative controls, while the wells containing kanamycin and nystatin (Taicheng Pharmaceutical Co., Guangdong, China, purity >99%) were used as the positive controls. Kanamycin showed antimicrobial activity against *B. subtilis* with MIC of 32 μg/mL. Nystatin showed antimicrobial activity against *N. oryzae*, *B. bassian*, and *M. albican* with MICs of 4 μg/mL, 4 μg/mL, and 16 μg/mL.

The NO inhibitory activity of these compounds was determined using the Griess reagent assay for NO production. Murine macrophage cell line was used as a detection model. The supernatants were used to measure the NO production with a 3-(4,5-dimethylthiazol-2-yl)-2,5-diphenyltetrazolium bromide (MTT) assay for cell viability. L-NMMA was used as the positive control.

AChE inhibitory activities of the compounds were assayed by the spectrophotometric method. S-acetylthiocholine iodide, *S*-butyrylthiocholine iodide, 5,5′-dithio-bis-(2-nitrobenzoic) acid (DTNB or Ellman’s reagent), and acetylcholinesterase, derived from human erythrocytes, were purchased from Sigma Chemical. The compounds were dissolved in DMSO. The reaction mixture (total volume of 200 μL) containing the phosphate buffer (pH = 8.0), a test compound (50 μM), and acetyl cholinesterase (0.02 U/mL), was incubated for 20 min at 37 °C. Then, the reaction was initiated by adding 40 μL of the solution containing DTNB (0.625 mM) and acetylthiocholine iodide (0.625 mM). The hydrolysis of acetylthiocholine was monitored at 405 nm every 30 s for an hour. Tacrine (Sigma, Darmstadt, Germany, purity >99%) was used as a positive control with an inhibition ratio of 52.63% and a final concentration of 0.333 μM. All the reactions were performed in triplicate. The percentage inhibition was calculated as follows: % inhibition = (E − S)/E × 100, where E is the activity of the enzyme without a test compound, and S is the activity of the enzyme with a test compound.

The cytotoxicity of metabolites **1** and **2** against tumor cells, HL-60, A-549, SMMC-7721, SW480, and MCF-7 were assessed in vitro by the 3-(4,5-dimethylthiazol-2-yl)-5(3-carboxymethoxyphenyl)-2-(4-sulfopheny)-2*H*-tetrazolium (MTS) means. The positive control of taxol was used with IC_50_ <0.008 μM.

Compounds were fully mixed with porcine pancreas lipase (PPL) solution, and *p*-nitrophenyl butyrate (*p*-NPB) was added to the mixture at 37 °C for 15 min. The optical density (OD) values were measured with a microplate reader at a wavelength of 400 nm. Orlistat was used as the positive control, with ratio of 94.906% at the concentration of 0.005 μM.

Each test compound was incubated with l-dopa (1.25 mM), and tyrosinase (25 U/mL) at room temperature for 5 min. The optical density (OD) values were measured with a microplate reader at a wavelength of 490 nm. Kojic acid was used as positive control agent, with ratio of 64.809% at a concentration of 10 μg/mL.

### 3.5. ECD Calculations

The theoretical calculations of these compounds were performed using the Gaussian Program by the Yunnan Electronic Computing Center. The geometries of the compounds were previously optimized by density functional theory (DFT) methods at the B3LYP/6-31G(d,p) level, and excitation energies and rotational strengths were calculated using time-dependent density functional theory (TDDFT) at the B3LYP/6-31G(d,p) level. The ECD spectrum was simulated from electronic excitation energies and velocity rotational strengths.

## Figures and Tables

**Table 1:** ^13^C NMR and ^1^H NMR data of compounds **1**–**3**.

Pos.	1		2		3	
	δ_H_	δ_c_	δ_H_	δ_c_	δ_H_	δ_c_
1	7.05 (s)	149.0	5.48 (s)	104.7	5.35 (s)	89.7
2						
3	4.76 (m)	75.6	4.56 (m)	74.3	4.68 (t, *J* = 6.0 Hz)	71.3
4	2.75, 3.03 (m)	33.5	2.56, 3.13 (m)	34.1	2.43 (dd, *J* = 2.5, 16.0 Hz) 3.12 (dd, *J* = 2.5, 15.5 Hz)	33.6
4a		149.7		151.9		156.0
5	5.58 (s)	115.6	5.95 (s)	127.2	5.96 (s)	124.7
6		201.0		195.1		198.0
7		78.3		75.2		75.0
8	4.57 (brs)	73.1	3.79 (brs)	75.8	3.81 (s)	73.4
8a		112.7		69.0		71.1
9	5.53 (dd, *J* = 6.0, 15.0 Hz)	125.8	4.56 (m)	77.0	5.69 (dd, *J* = 6.5 Hz, 15.5 Hz)	128.1
10	6.25 (dd, *J* = 10.5, 15.0 Hz)	133.6	2.75 (d, *J* = 6.0 Hz)	48.0	6.27 (dd, *J* = 10.5, 15.5 Hz)	133.9
11	6.02 (m)	130.2	9.74 (t, *J* = 1.6 Hz)	199.8	6.07 (m)	131.0
12	5.77 (m)	132.0	1.34 (s)	19.4	5.77 (m)	130.3
13	1.77 (d, *J* = 6.5 Hz)	18.1			1.76 (d, *J* = 5.5 Hz)	16.8
14	1.18 (s)	18.8			1.37 (s)	18.9

**Table 2:** ^13^C NMR and ^1^H NMR data of compounds **4** and **5**.

Pos.	4		5	
8	δ_H_	δ_c_	δ_H_	δ_c_
1	6.96 (s)	147.1	5.67 (s)	87.6
2				
3	4.60 (t, *J* = 6.0 Hz)	80.4	4.34 (m)	66.1
4	4.24 (d, *J* = 6.0 Hz)	67.4	1.97, 2.15 (m)	27.3
4a		150.6		129.2
5	5.77 (s)	115.1		198.4
6		201.7	3.99 (s)	79.6
7		77.2		79.5
8	4.41 (brs)	73.1	4.43 (t, *J* = 2.7 Hz)	73.7
8a		111.8		152.1
9	5.51 (dd, *J* = 6.0, 15.0 Hz)	124.0	5.51 (dd, *J* = 6.0, 15.0 Hz)	129.5
10	6.31 (dd, *J* = 10.5, 15.5 Hz)	134.2	6.18 (dd, *J* = 10. 2, 15.6 Hz)	131.6
11	6.07 (m)	130.7	5.97 (m)	130.8
12	5.78 (m)	130.4	5.62 (m)	129.6
13	1.76 (d, *J* = 5.0 Hz)	16.8	1.66 (d, *J* = 6.6 Hz)	16.8
14	1.20 (s)	17.7	0.92 (s)	12.6

## Supplementary Materials

The following are available online. 1D, 2D NMR, MS and CD of the new compounds **1**–**5**, and the plate test (Figures S1–S32).
