# Supplementary material for: New Azaphilones from Nigrospora oryzae Co-Cultured with Beauveria bassiana
Source: Molecules. 2018 Jul 21;23(7):1816. doi: 10.3390/molecules23071816 (PMC6100547; doi:10.3390/molecules23071816)
Supplement: Supplementary file 1 [file molecules-23-01816-s001.pdf]

Supplementary Materials

New Azaphilones from *Nigrospora oryzae* Co-Cultured with *Beauveria bassiana*

Zhuo-Xi Zhang[1], Xue-Qiong Yang[1], Qing-Yan Zhou, Bang-Yan Wang, Ming Hu, Ya-Bin Yang*, Hao Zhou, and Zhong-Tao Ding*

Functional Molecules Analysis and Biotransformation Key Laboratory of Universities in Yunnan Province, School of Chemical Science and Technology, Yunnan University, 2st Cuihu North Road, Kunming 650091, China;

Corresponding authors:

Doctor Ya-Bin Yang, School of Chemical Science and Technology, Yunnan University, Kunming 650091, China. E-mail: ybyang@ynu.edu.cn

Professor Zhong-Tao Ding, School of Chemical Science and Technology, Yunnan University, Kunming 650091, China. E-mail: ztding@ynu.edu.cn

[1]These authors contributed equally to this work.

Table of contents

Figure S1. HRESIMS spectrum of compound **1** 3
Figure S2. ^{1}H NMR spectrum of compound **1** in $CDCl_3$ (500 MHz) 3
Figure S3. ^{13}C NMR spectrum of compound **1** in $CDCl_3$ (125MHz) 4
Figure S4. COSY spectrum of compound **1** in $CDCl_3$ (500 MHz) 4
Figure S5. HSQC spectrum of compound **1** in $CDCl_3$ (500 MHz) 5
Figure S6. HMBC spectrum of compound **1** in $CDCl_3$ (500 MHz) 5
Figure S7. NOESY spectrum of compound **1** in $CDCl_3$ (500 MHz) 6
Figure S8. HRESIMS spectrum of compound **2** 6
Figure S9. ^{1}H NMR spectrum of compound **2** in acetone (600 MHz) 7
Figure S10. ^{13}C NMR spectrum of compound **2** in acetone (150MHz) 7
Figure S11. COSY spectrum of compound **2** in acetone (600MHz) 8
Figure S12. HSQC spectrum of compound **2** in acetone (600MHz) 8
Figure S13. HMBC spectrum of compound **2** in acetone (600MHz) 9
Figure S14. NOESY spectrum of compound **2** in acetone (600MHz) 9
Figure S15.HRESIMS spectrum of compounds **3** and **4** 10
Figure S16. ^{1}H NMR spectrum of compounds **3** and **4** in MeOD (500 MHz) 10
Figure S17. ^{13}C NMR spectrum of compounds **3** and **4** in MeOD (125 MHz) 11
Figure S18. COSY spectrum of compounds **3** and **4** in MeOD (500MHz) 11
Figure S19. HSQC spectrum of compounds **3** and **4** in MeOD (500MHz) 12
Figure S20. HMBC spectrum of compounds **3** and **4** in MeOD (500MHz) 12
Figure S21. NOESY spectrum of compounds **3** and **4** in MeOD (500MHz) 13
Figure S22.HRESIMS spectrum of compound **5** 13
Figure S23. ^{1}H NMR spectrum of compound **5** in MeOD (600 MHz) 14
Figure S24. ^{13}C NMR spectrum of compound **5** in MeOD (150MHz) 14
Figure S25. COSY spectrum of compound **5** in MeOD (600MHz) 15
Figure S26. HSQC spectrum of compound **5** in MeOD (600MHz) 15
Figure S27. HMBC spectrum of compound **5** in MeOD (600MHz) 16
Figure S28.NOESY spectrum of compound **5** in MeOD (600MHz) 16
Figure S29. CD spectrum of compound **1** 17
Figure S30.CD spectrum of compounds **2** 17
Figure S31.CD spectrum of compounds **5** 18
Figure S32. The plate culture of *Beauveria bassiana,* and *Nigrospora oryzae* in 3 days (Sequenced as the left to right). 18

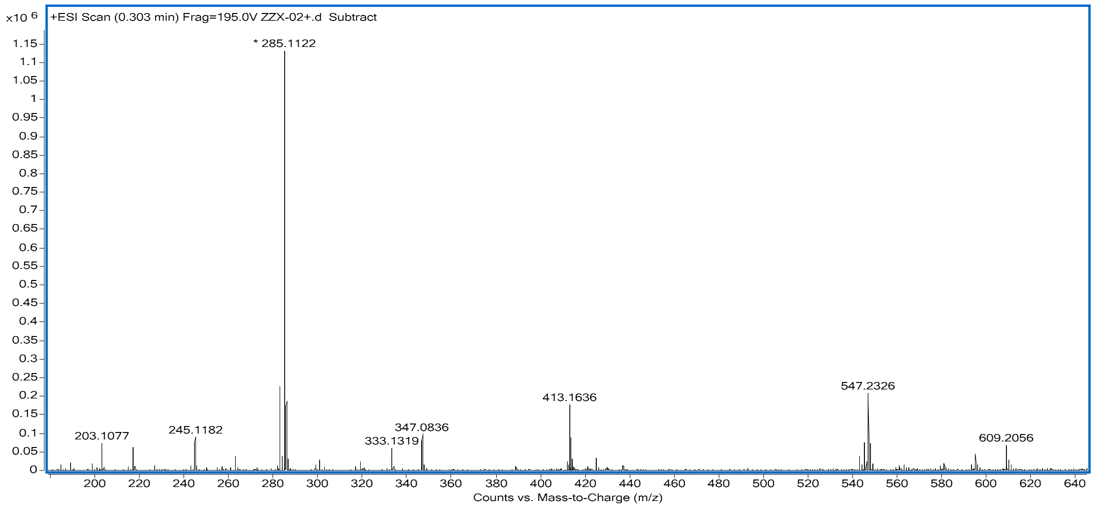

Figure S1. HRESIMS spectrum of compound **1**

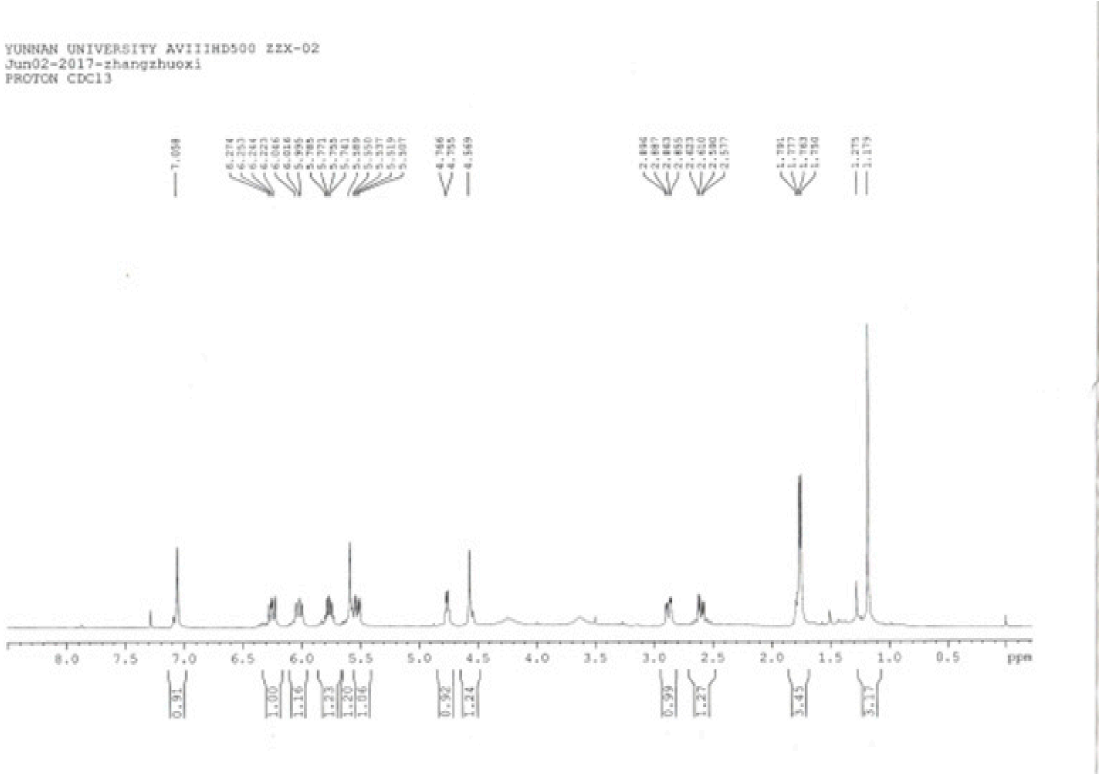

Figure S2. ^{1}H NMR spectrum of compound **1** in $CDCl_3$ (500 MHz)

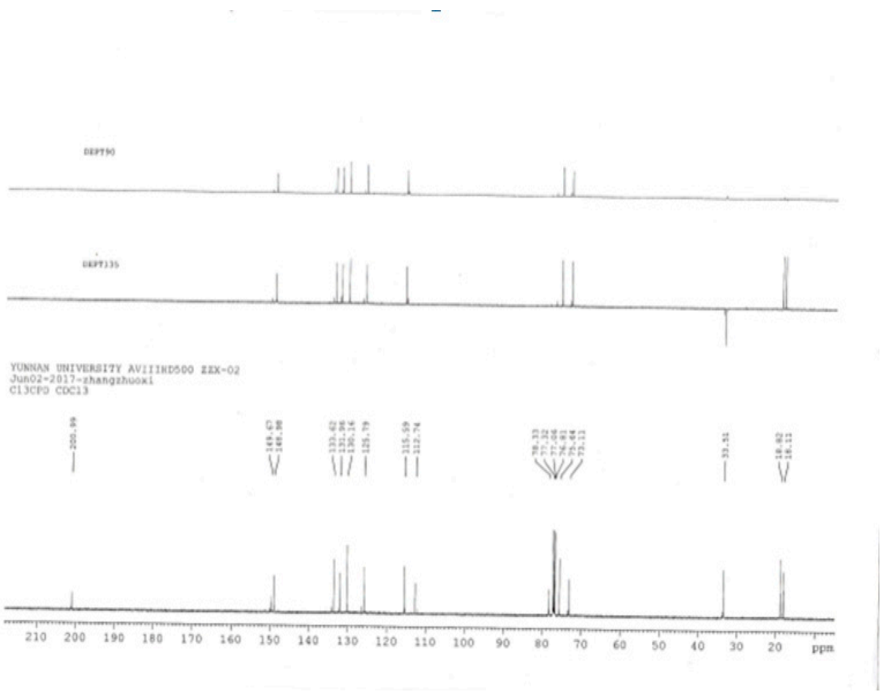

Figure S3. ^{13}C NMR spectrum of compound **1** in $CDCl_3$ (125MHz)

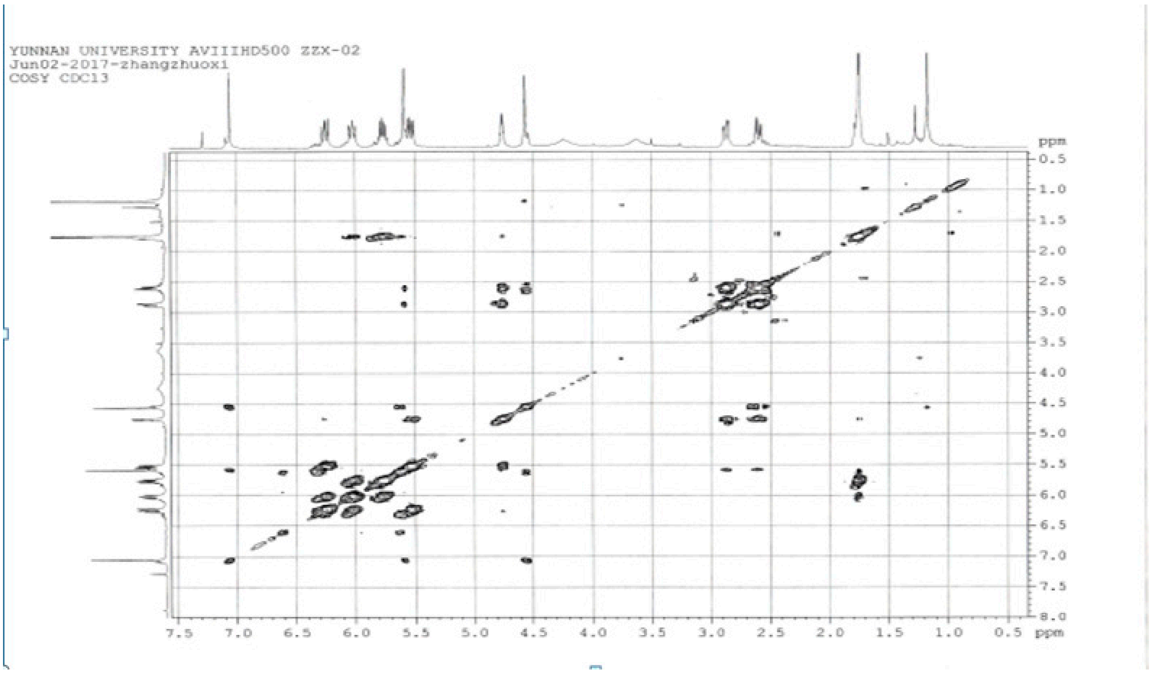

Figure S4. COSY spectrum of compound **1** in $CDCl_3$ (500 MHz)

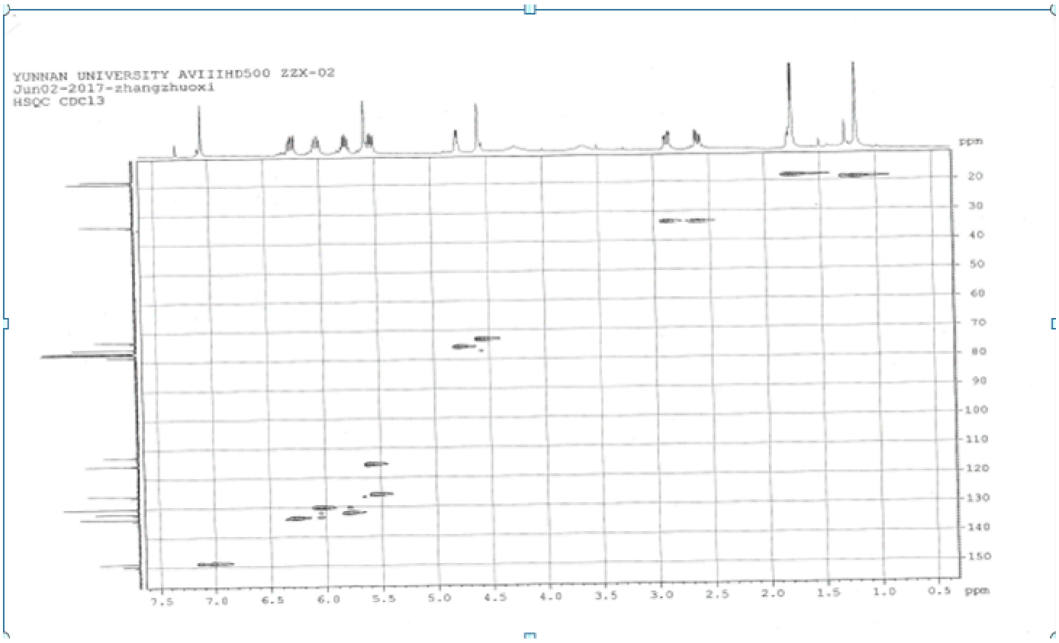

Figure S5. HSQC spectrum of compound **1** in $CDCl_3$ (500 MHz)

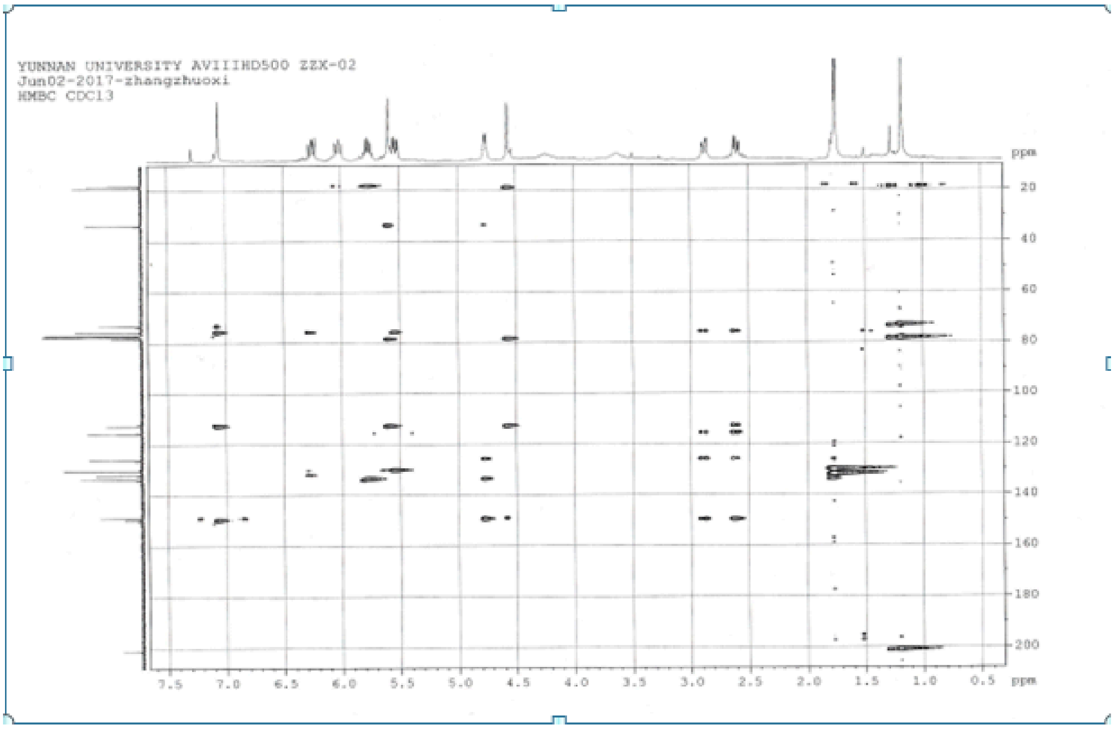

Figure S6. HMBC spectrum of compound **1** in $CDCl_3$ (500 MHz)

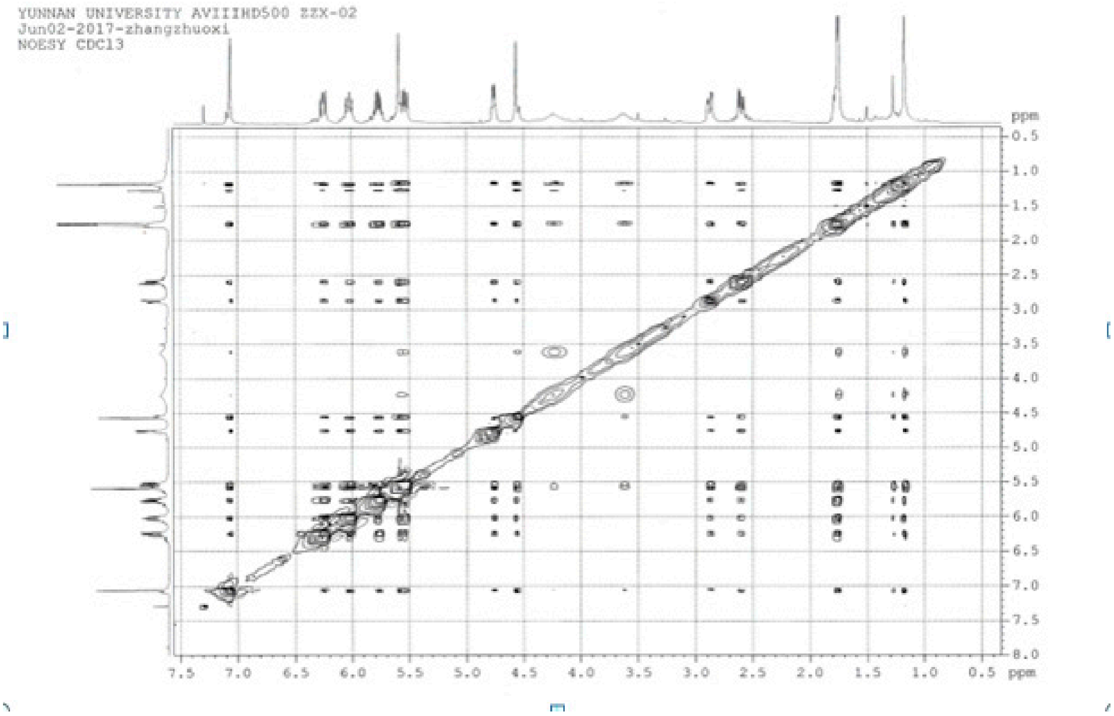

Figure S7. NOESY spectrum of compound **1** in $CDCl_3$ (500 MHz)

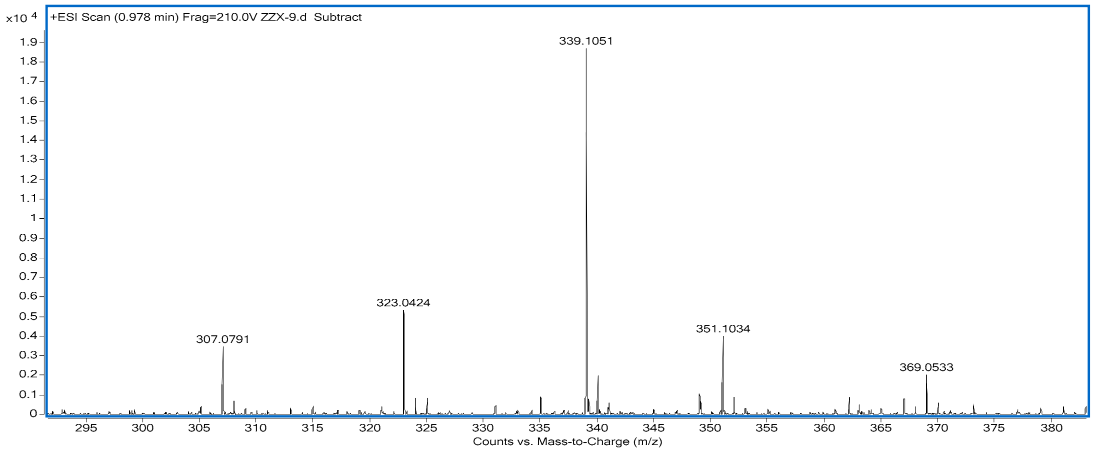

Figure S8. HRESIMS spectrum of compound **2**

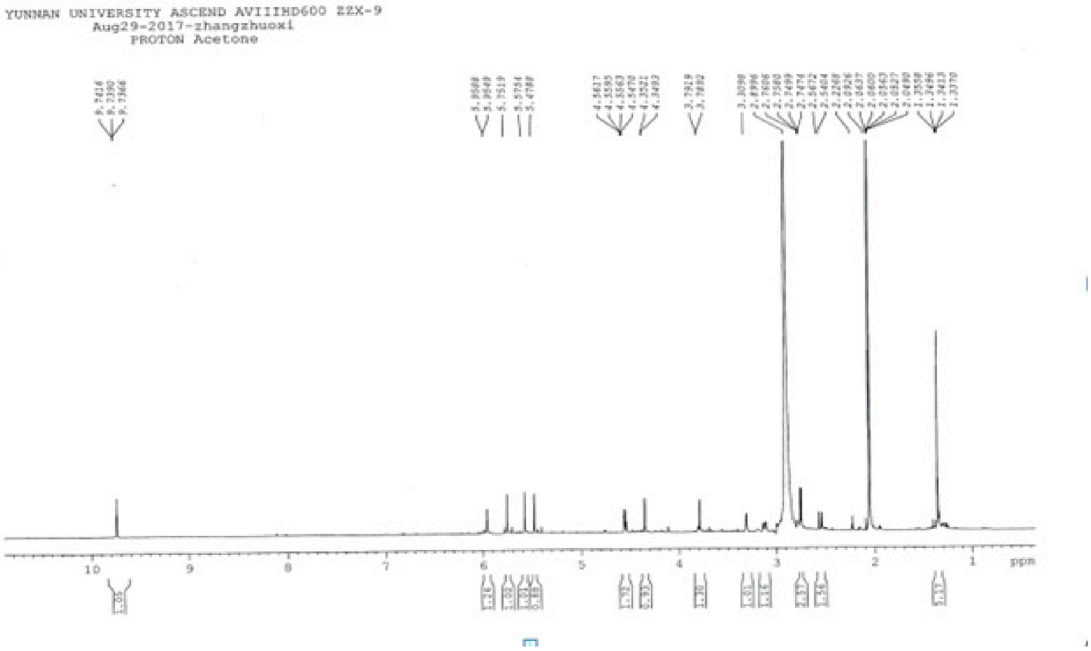

Figure S9. ^{1}H NMR spectrum of compound **2** in acetone (600 MHz)

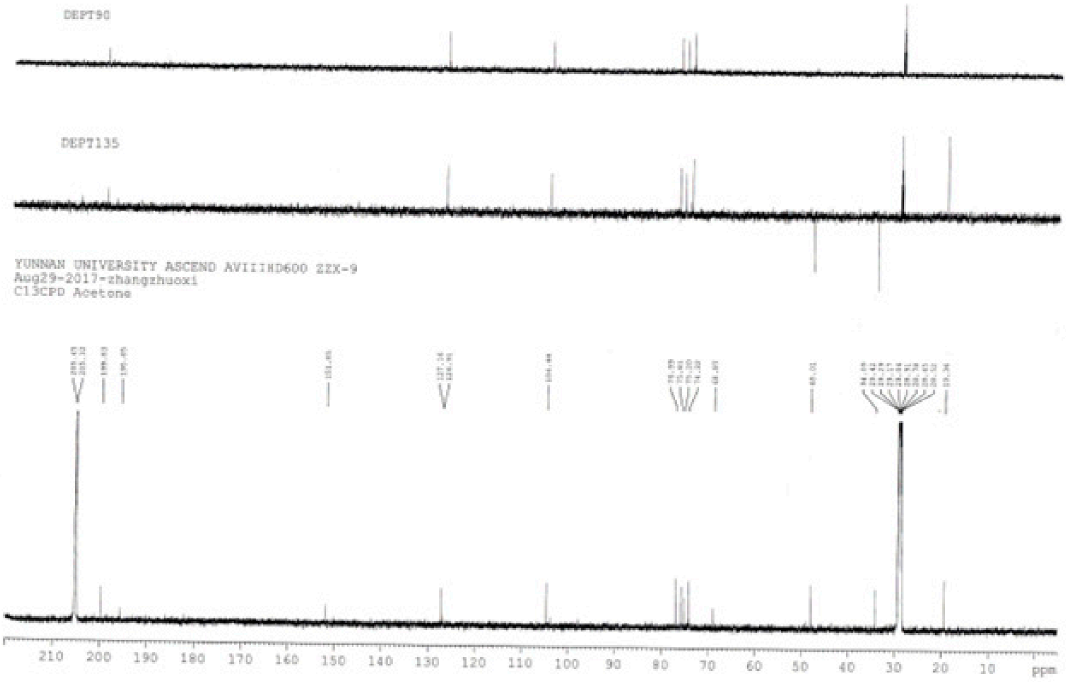

Figure S10. ^{13}C NMR spectrum of compound **2** in acetone (150MHz)

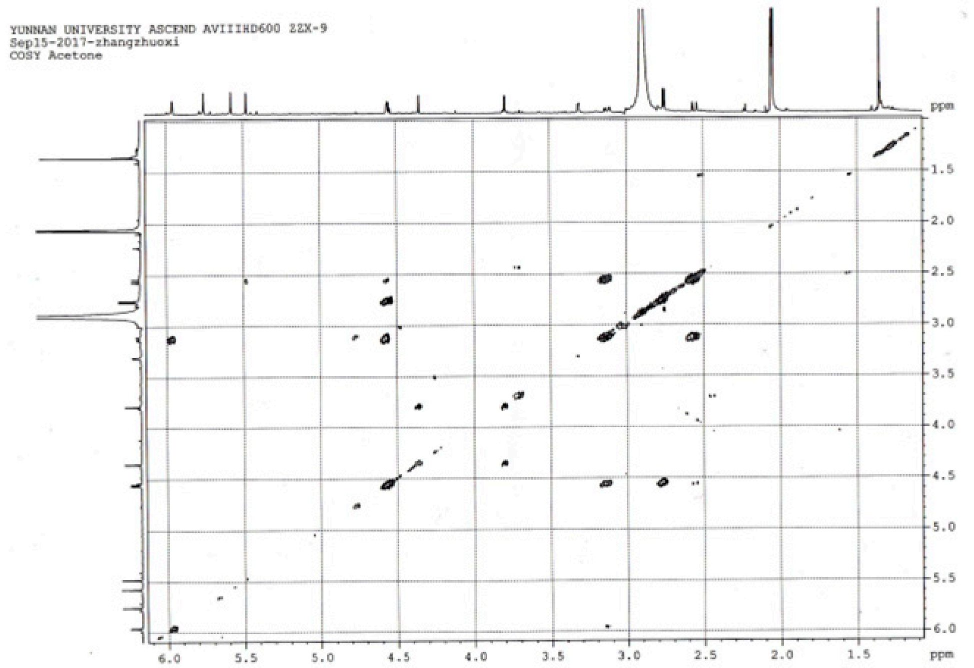

Figure S11. COSY spectrum of compound **2** in acetone (600MHz)

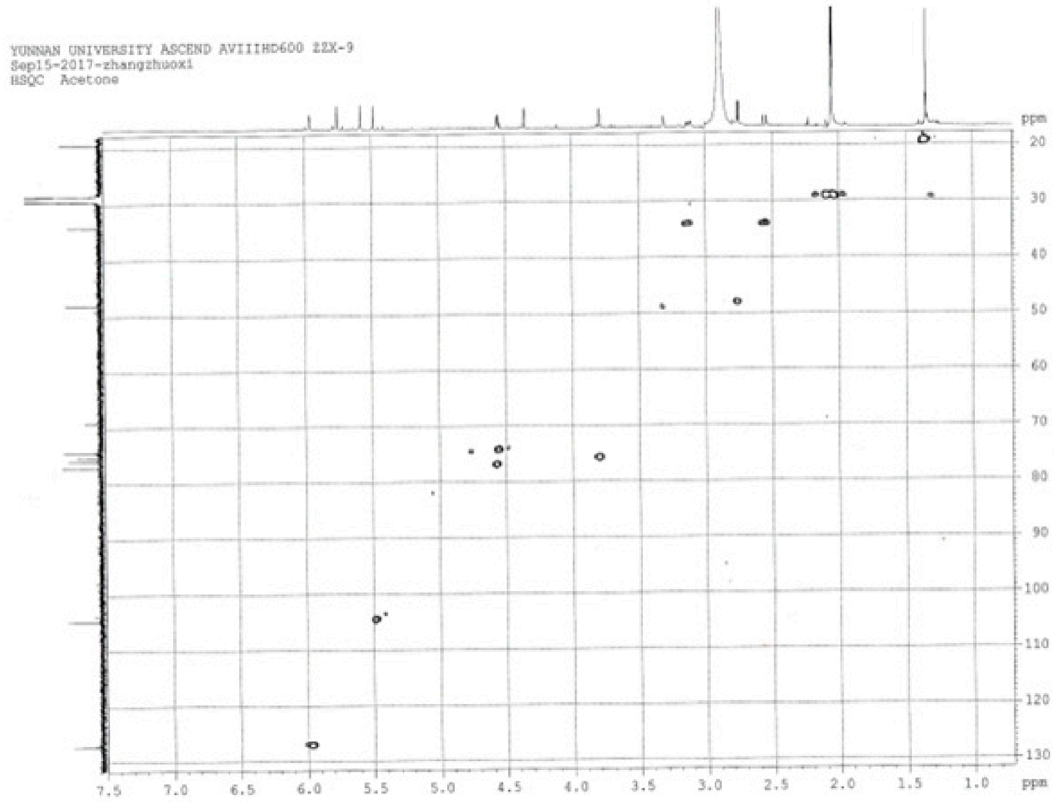

Figure S12. HSQC spectrum of compound **2** in acetone (600MHz)

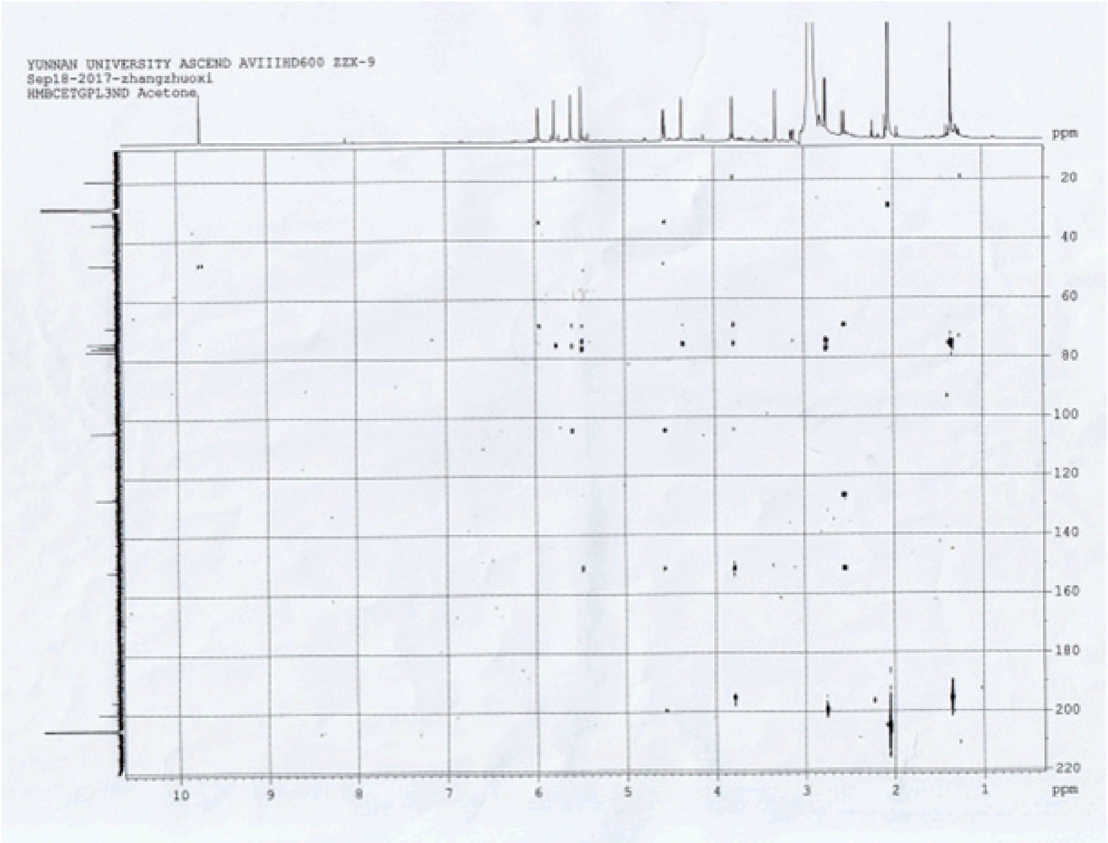

Figure S13. HMBC spectrum of compound **2** in acetone (600MHz)

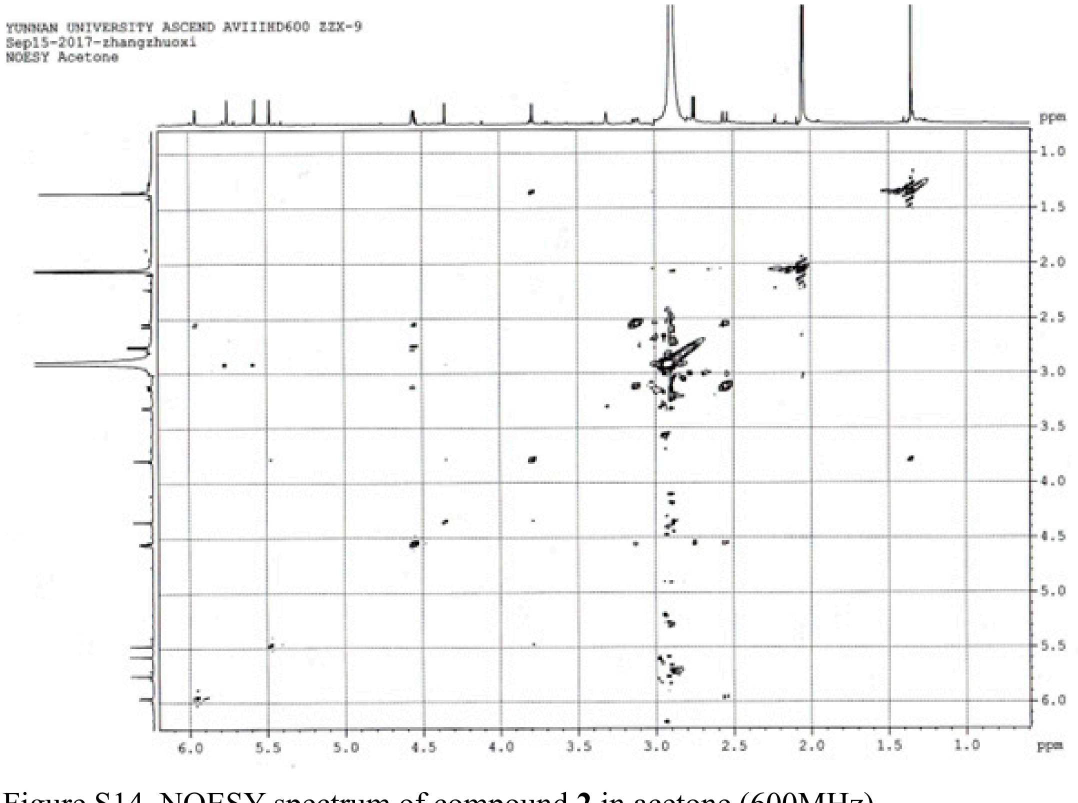

Figure S14. NOESY spectrum of compound **2** in acetone (600MHz)

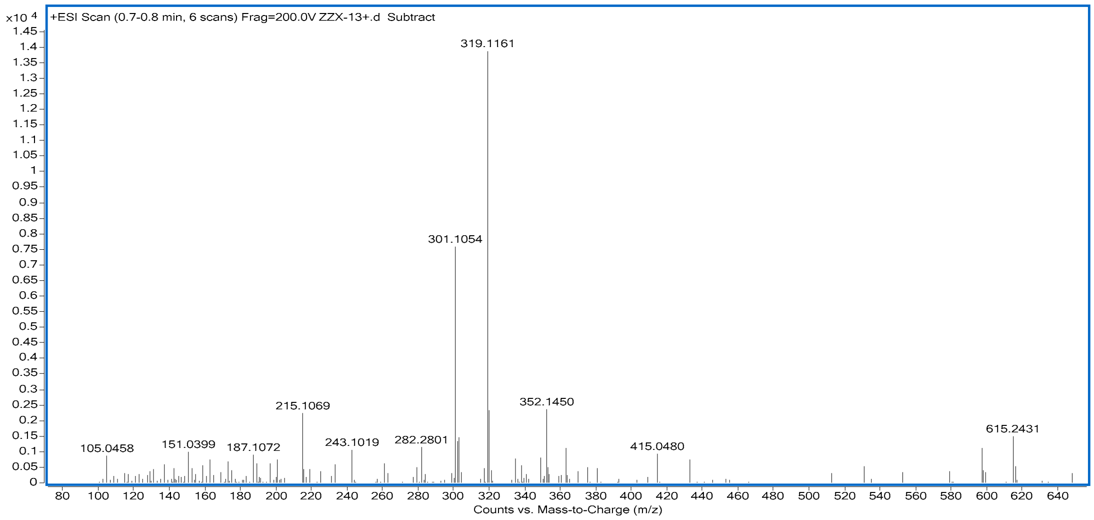

Figure S15.HRESIMS spectrum of compounds **3** and **4**

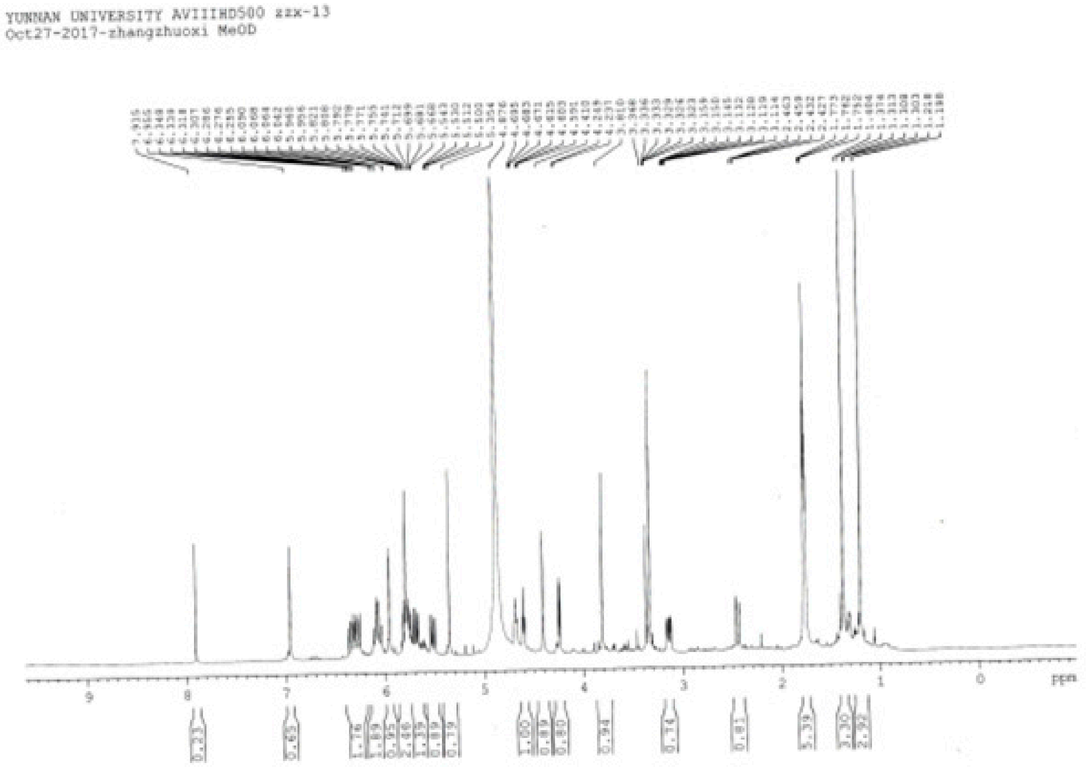

Figure S16. ^{1}H NMR spectrum of compounds **3** and **4** in MeOD (500 MHz)

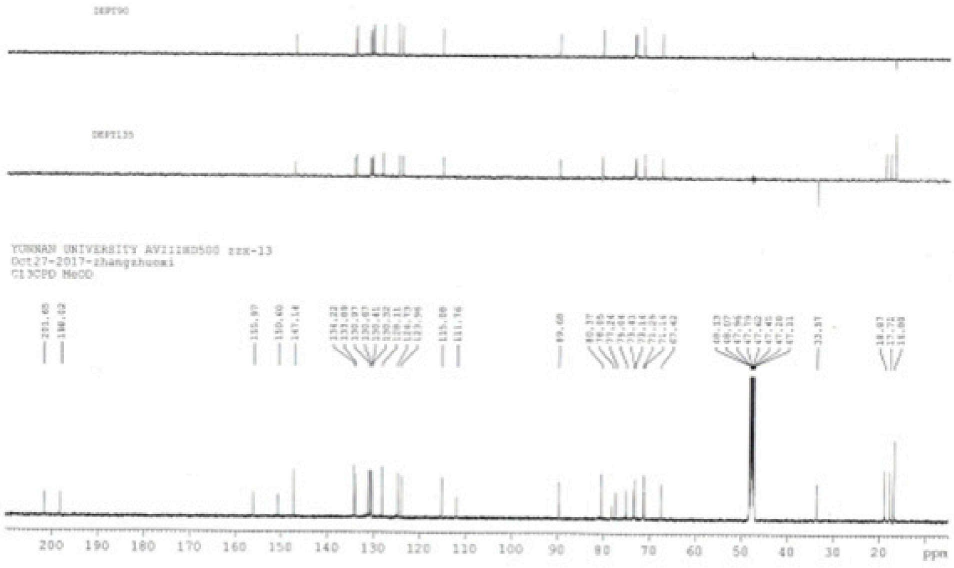

Figure S17. ^{13}C NMR spectrum of compounds **3** and **4** in MeOD (125 MHz)

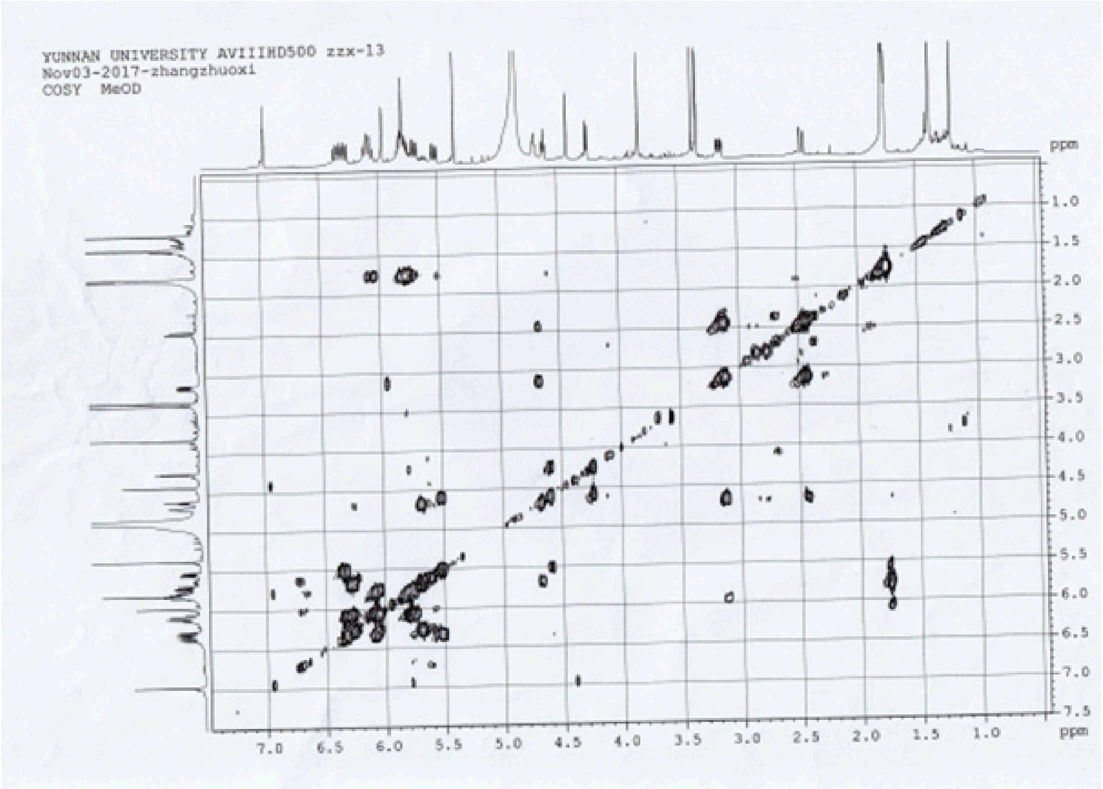

Figure S18. COSY spectrum of compounds **3** and **4** in MeOD (500MHz)

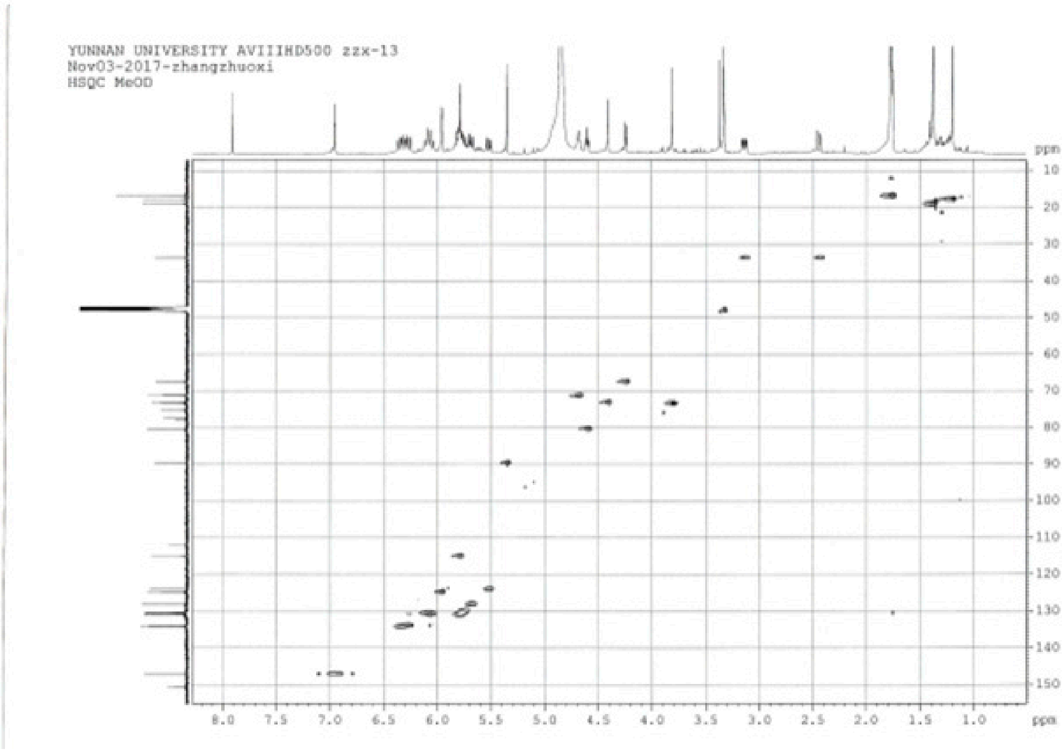

Figure S19. HSQC spectrum of compound **3** and **4** in MeOD (500MHz)

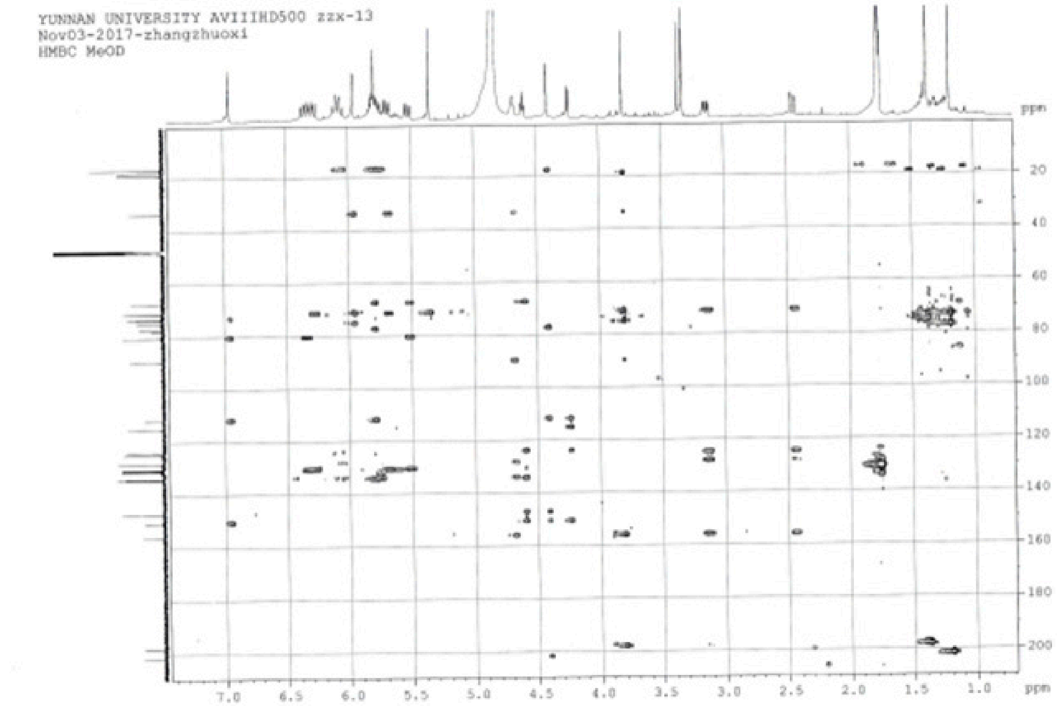

Figure S20. HMBC spectrum of compounds **3** and **4** in MeOD (500MHz)

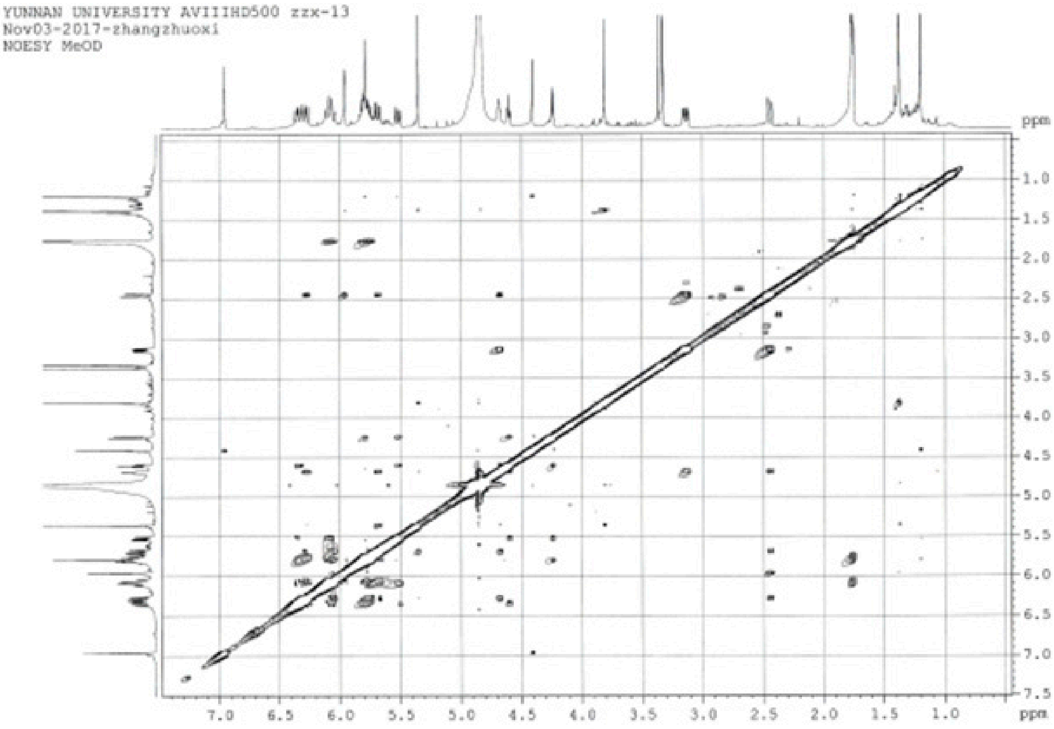

Figure S21. NOESY spectrum of compounds **3** and **4** in MeOD (500MHz)

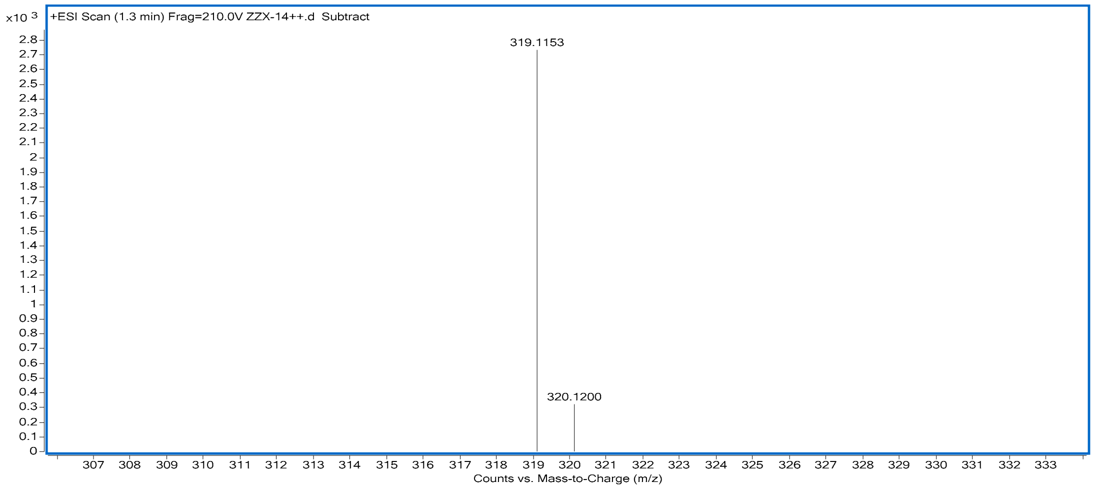

Figure S22.HRESIMS spectrum of compound **5**

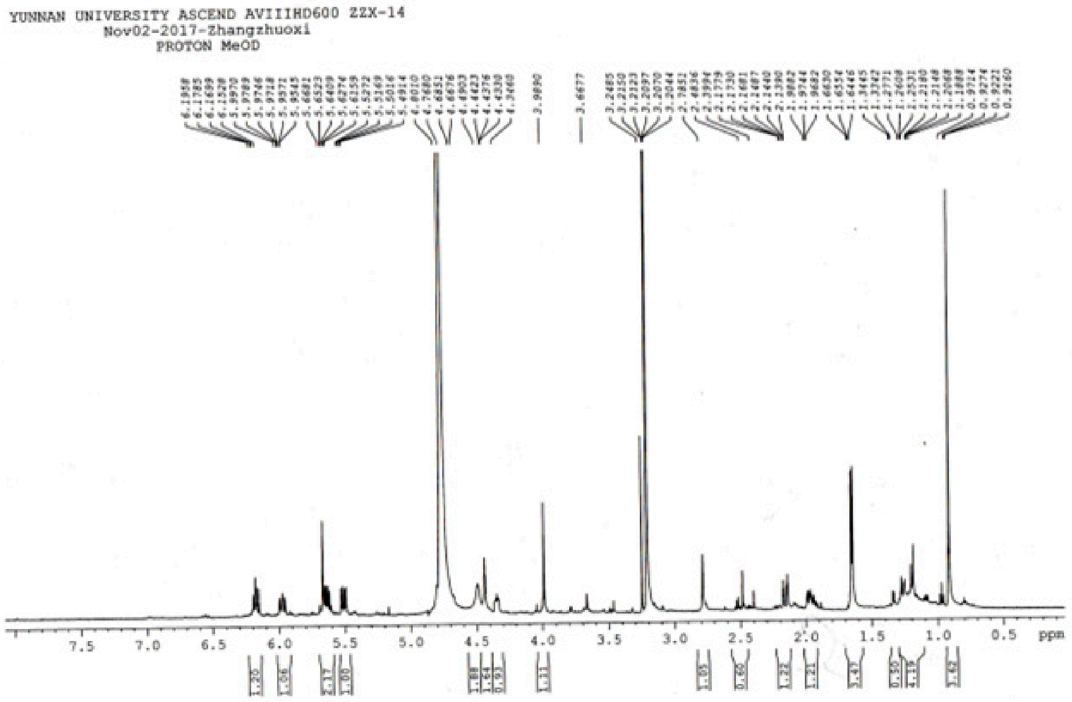

Figure S23. ^{1}H NMR spectrum of compound **5** in MeOD (600 MHz)

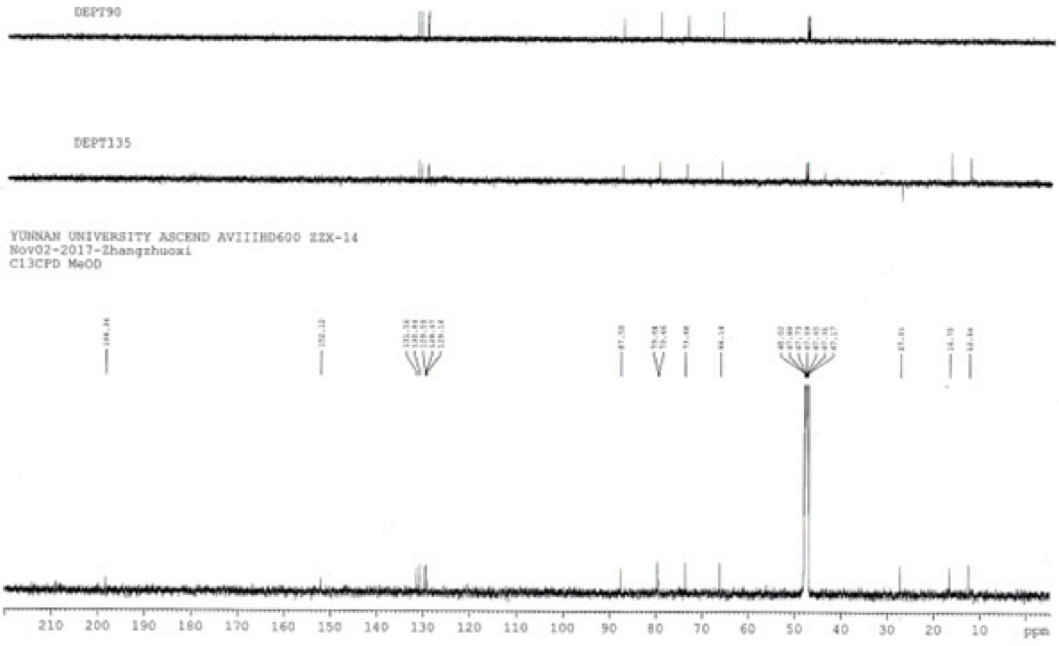

Figure S24. ^{13}C NMR spectrum of compound **5** in MeOD (150MHz)

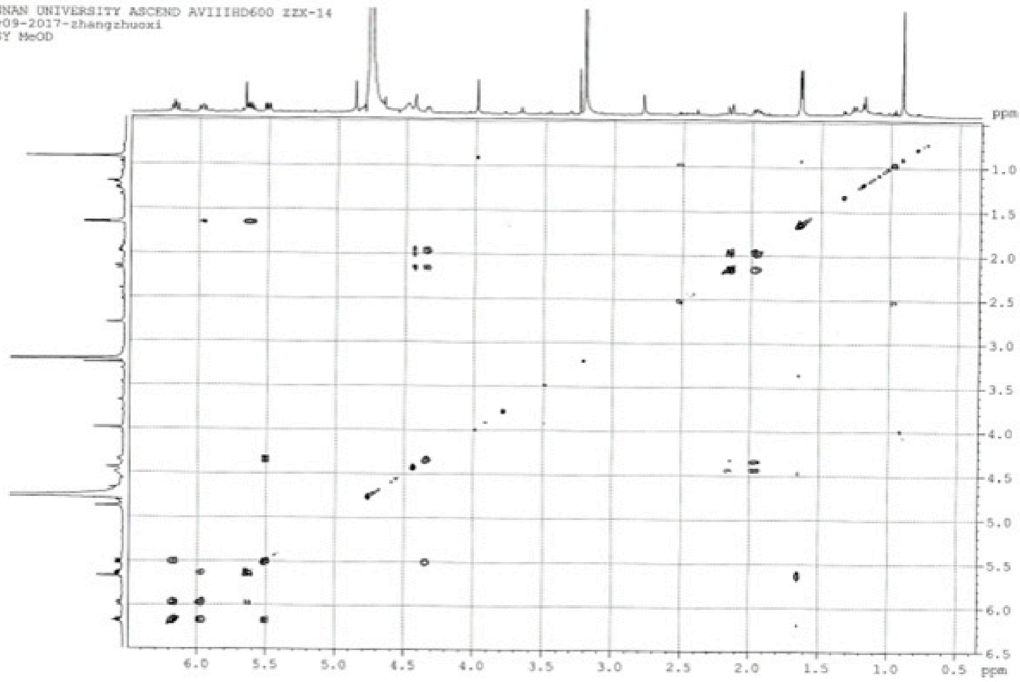

Figure S25. COSY spectrum of compound **5** in MeOD (600MHz)

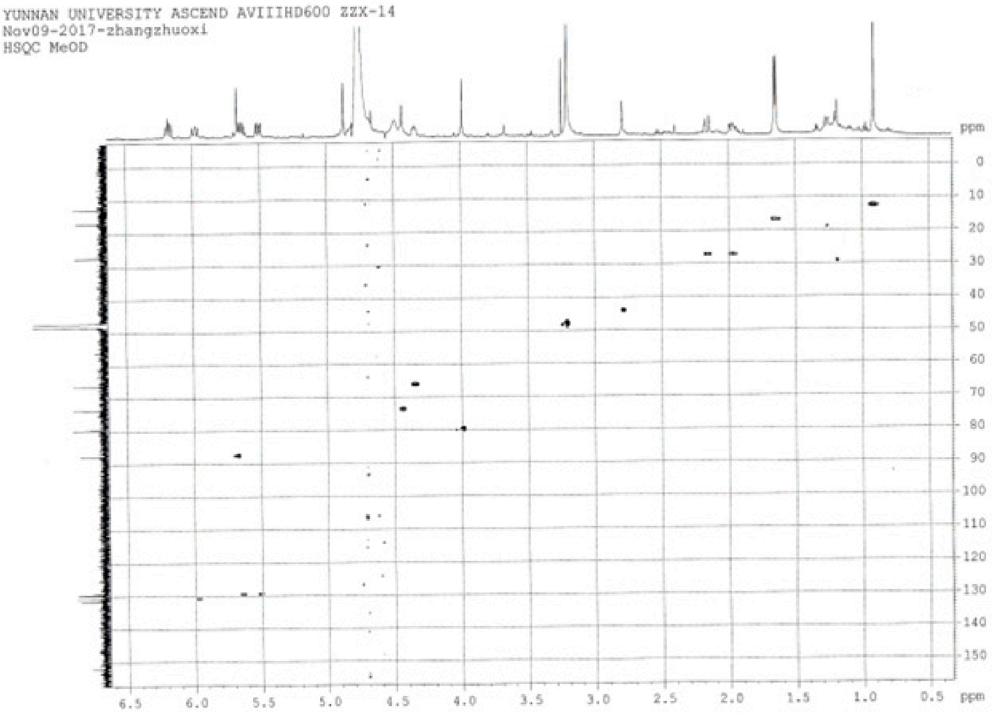

Figure S26. HSQC spectrum of compound **5** in MeOD (600MHz)

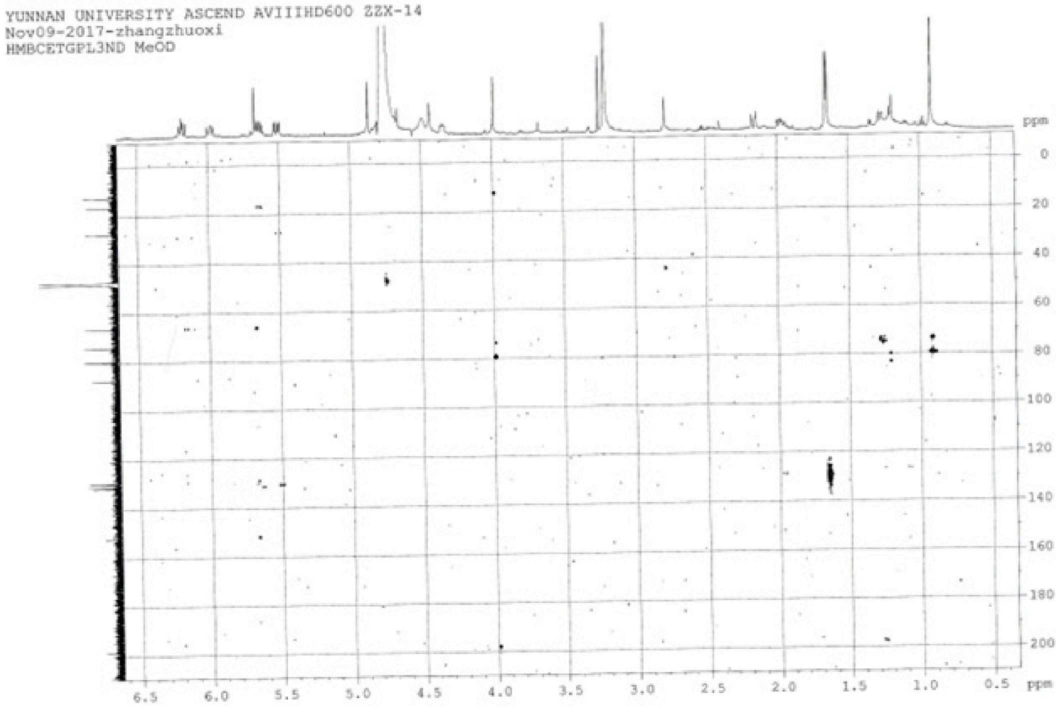

Figure S27. HMBC spectrum of compound **5** in MeOD (600MHz)

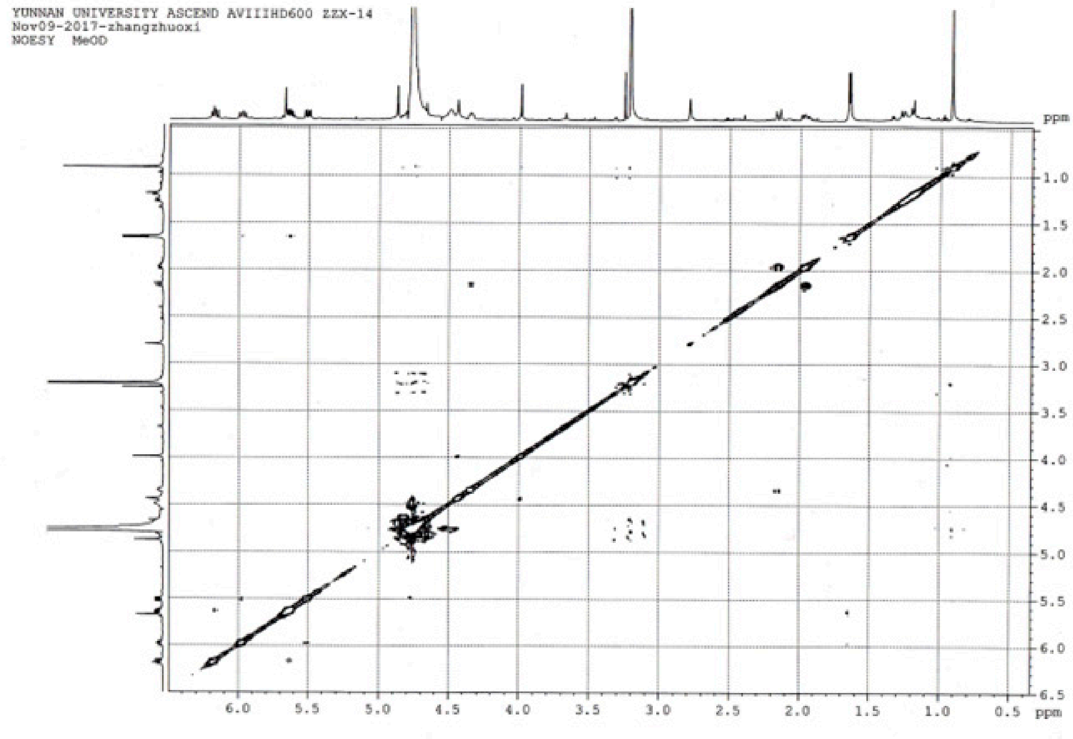

Figure S28.NOESY spectrum of compound **5** in MeOD (600MHz)

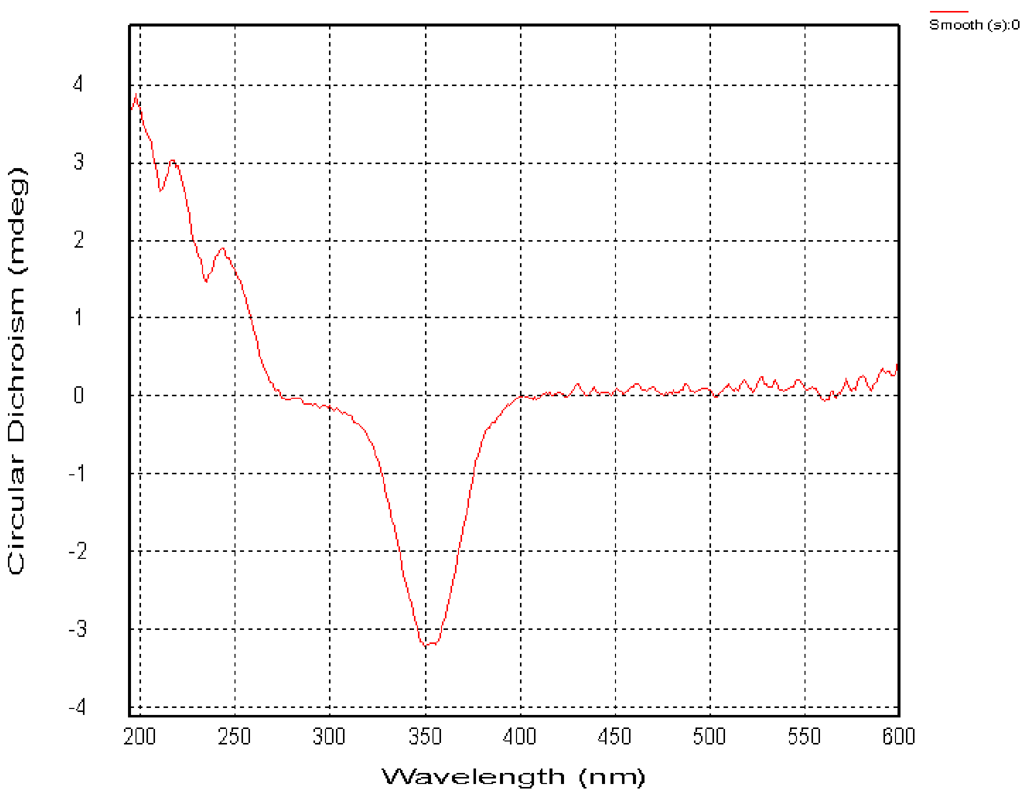

Figure S29. CD spectrum of compound **1**

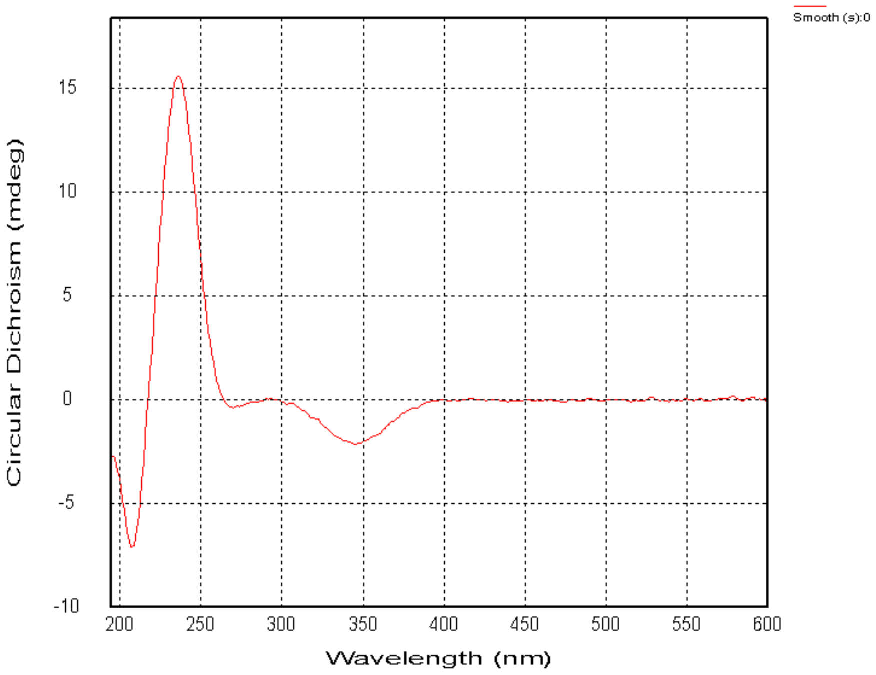

Figure S30. CD spectrum of compound **2**

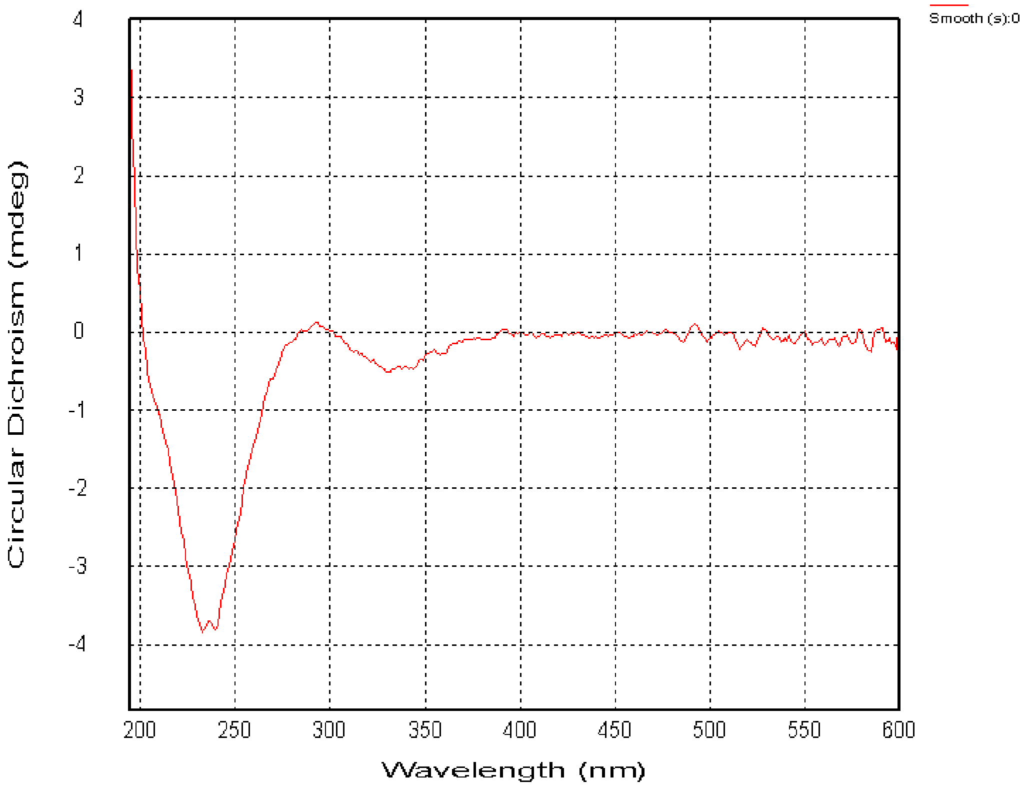

Figure S31. CD spectrum of compound **5**

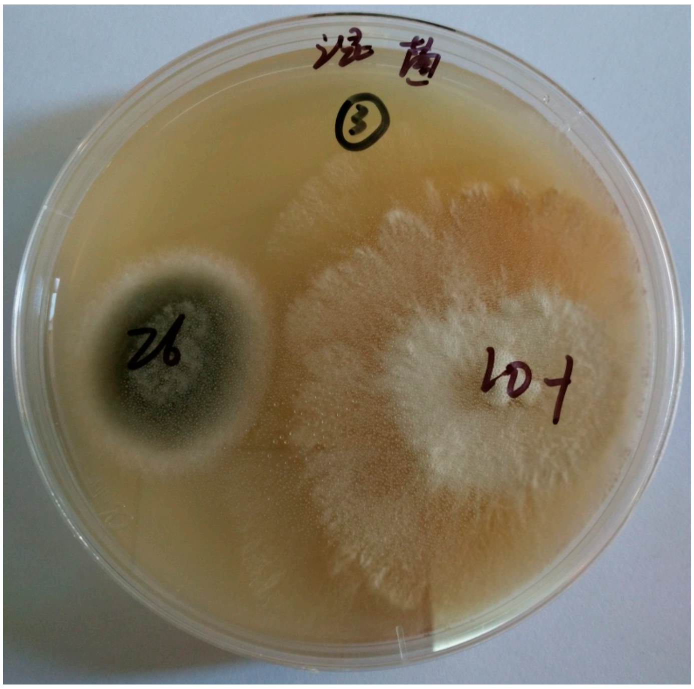

Figure S32. The plate culture of *Beauveria bassiana,* and *Nigrospora oryzae* in 3 days (Sequenced as the left to right).
